# Perspective of an International Online Patient and Caregiver Community on the Burden of Spasticity and Impact of Botulinum Neurotoxin Therapy: Survey Study

**DOI:** 10.2196/17928

**Published:** 2020-12-07

**Authors:** Atul T Patel, Theodore Wein, Laxman B Bahroo, Ophélie Wilczynski, Carl D Rios, Manuel Murie-Fernández

**Affiliations:** 1 Kansas City Bone & Joint Clinic Overland Park, KS United States; 2 Department of Neurology and Neurosurgery McGill University Montreal, QC Canada; 3 Department of Neurology Georgetown University Georgetown, DC United States; 4 Carenity Paris France; 5 Ipsen Pharma Boulogne-Billancourt France; 6 Neurorehabilitation Unit Ciudad de Telde Hospital Las Palmas Spain

**Keywords:** spasticity, activities of daily living, quality of life, survey methodology

## Abstract

**Background:**

Patient- and caregiver-reported data are lacking on the burden of spasticity, and the impact of botulinum neurotoxin type A (BoNT-A) treatment for this condition, on patients' daily lives. As recommended in recent guidance from the US Food and Drug Administration, online patient communities can represent a platform from which to gather specific information outside of a clinical trial setting on the burden of conditions experienced by patients and caregivers and their views on treatment options in order to inform evidence-based medicine and drug development.

**Objective:**

The objective of our study is to characterize spasticity symptoms and their associated burdens on Western European and US patients and caregivers in the realms of work, daily activities, quality of life (QoL), as well as the positive and negative impacts of treatment with BoNT-A (cost, time, QoL) using Carenity, an international online community for people with chronic health conditions.

**Methods:**

We performed a noninterventional, multinational survey. Eligible participants were 18 years old or older and had, or had cared for, someone with spasticity who had been treated with BoNT-A for at least 1 year. Patients and caregivers were asked to complete an internet-based survey via Carenity; caregivers reported their own answers and answered on behalf of their patients. Questions included the burden of spasticity on the ability to work, functioning, daily-living activities, and QoL, the impact of BoNT A therapy on patients' lives, and the potential benefits of fewer injections.

**Results:**

There were 615 respondents (427 patients and 188 caregivers). The mean age of patients and caregivers was 41.7 years and 38.6 years, respectively, and the most commonly reported cause of spasticity was multiple sclerosis. Caregivers were most often the parents (76/188, 40%) or another family member (51/188, 27%) of their patients. Spasticity had a clear impact on patients' and caregivers' lives, including the ability to work and injection costs. For patients, spasticity caused difficulties with activities of daily living and reduced QoL indices. The median number of BoNT-A injections was 4 times per year, and 92% (393/427) of patients reported that treatment improved their overall satisfaction with life. Regarding the BoNT-A injection burden, the greatest patient-reported challenges were the cost and availability of timely appointments. Overall, 86% (368/427) of patients believed that a reduced injection frequency would be beneficial. Caregivers answering for their patients gave largely similar responses to those reported by patients.

**Conclusions:**

Spasticity has a negative impact on both patients' and caregivers' lives. All respondents reported that BoNT A treatment improved their lives, despite the associated challenges. Patients believed that reducing the frequency of BoNT-A injections could alleviate practical issues associated with treatment, implying that a longer-acting BoNT-A injection would be well received.

## Introduction

Knowledge of patient and caregiver perspectives on a disease and related therapies help identify relevant patient-reported outcomes for assessment in clinical trials and inform clinical decision-making; therefore, it is critical for patient-focused drug development. Methodological guidance to support the collection of patient experience data has recently been published by the US Food and Drug Administration (FDA), which includes the utilization of online patient communities [1]. An increasing number of internet-based platforms are available to patients for their participation in scientific research, ranging from registry data, forums, social networks, and online communities. Online patient communities offer the opportunity to voluntarily express experiences and feelings outside of the clinical setting regarding the treatments for their condition and the burden on their quality of life (QoL) [2]. These data, along with any epidemiological information, can provide researchers with a better understanding of the patient journey for a given disease and are important in clinical judgment and decision making for evidence-based medicine [2]. The internet provides a vast potential resource for real-world data collection during scientific studies, which, along with exploring patient expectations and unmet needs, may help develop or support study protocols and methodologies and enhance recruitment for research. Developed in 2011, Carenity is an international online patient community for people with chronic conditions [3]. Currently, 500,000 members are registered on the platform, which allows both patients and their families to share their experiences, follow the evolution of their health, and contribute to medical research through online surveys.

Spasticity is caused by an upper motor neuron lesion leading to intermittent or sustained involuntary activation of muscles [4] and is a sequela from a range of central nervous system (CNS) disorders affecting over 12 million people worldwide [5]. The prevalence of spasticity differs between etiologies, which include stroke (40%), multiple sclerosis (MS, 80%), spinal cord (65%) or traumatic brain (17-50%) injury, and cerebral palsy (90%) [6-11]. Left untreated, spasticity becomes burdensome both physically and economically for patients and their caregivers. Pain, spasms, limb contracture, and deformity can be experienced by patients with spasticity, leading to impairment of dexterity, mobility, and self-care, and ultimately, to decreased functioning and participation [12,13]. In an international survey of 281 patients with spasticity, 72% reported a negative impact on QoL and 44% reported a loss of independence [14]. Most respondents (64%) were cared for by family members, approximately 50% of whom had to reduce work hours or stop working in order to be a caregiver [14]. Spasticity also places an economic burden on patients, caregivers, and health care systems [14-16].

Treatment of spasticity is indicated when it interferes with function or QoL. As spasticity can change over time, patients should undergo continuous re-evaluation [13]. Treatment options for spasticity include physical and pharmacological therapies, as well as surgery in severe or intractable cases [13]. In addition to relieving symptoms, treatment aims to improve patients' functioning. Specifically, spasticity management should focus on achieving the patient's goals and the goals of caregivers and health care providers [13]. These may include goals associated with moving and walking, self-care, pain, changing and maintaining body positions, improving positions to participate in rehabilitation, and enabling orthotic use [17].

Botulinum neurotoxin type A (BoNT-A) is integral to focal and multifocal spasticity management [13] and has proven antispastic efficacy in stroke [18], CNS lesions [19,20], MS [21], and cerebral palsy [22,23]. Although recent studies have demonstrated improvements in active function following repeated injection cycles with abobotulinumtoxinA (aboBoNT-A) in adult patients with upper and lower limb spasticity [24,25], more evidence is needed to document functional improvements following BoNT-A treatment [18,26,27]. Historically, clinical studies have not recorded patient and caregiver perspectives on disease burden; thus, limited information is currently available on the impact of attending appointments, receiving BoNT-A injections, and the therapeutic outcomes of BoNT-A treatment on the daily lives of patients and their caregivers. In the only study published to date on this topic, caregiver burden has been shown to lessen with BoNT-A treatment for spasticity [28]. In another study in patients with dystonia who received BoNT treatment, the related caregiver burden appeared to be low but greater in those caring for patients who had more severe symptoms that had a greater impact on health-related QoL [29].

The aim of this study is to characterize spasticity symptoms and understand their burden, as well as the impact of BoNT-A injections, from the patient and caregiver perspective. The ability to work, perform daily activities, and QoL were assessed, as well as the perceived benefits and challenges associated with BoNT-A treatment with a focus on injection frequency.

## Methods

### Survey Design

The survey was conducted in France, Germany, Italy, Spain, the United Kingdom, and the United States between November 10, 2017, and February 28, 2018, using an established online approach [30-32]. Patients or caregivers of patients with spasticity who were members of the Carenity platform were invited by email to complete a questionnaire, presented in the local language, translated by a specialized agency and reviewed by local Carenity community managers. The study methodology and questionnaire were validated by 3 neurologists (from Canada, Spain, and the United States) and 1 rehabilitation physician (from the United States). To ensure language suitability, a patient who matched the study inclusion criteria was selected via the Carenity platform and asked to proofread the questionnaire.

All questions were the same between countries except for those relating to BoNT-A formulation brand names, which were removed from the Spanish questionnaire to comply with Spanish regulations. The study had a prespecified target of 600 respondents: included 300 from the United States and 300 from Europe, determined using the Cochran formula, with *p* set at 0.5 (maximum variability), the confidence level at 95%, and a maximum margin of error between 5% and 6% for each region.

### Survey Questionnaire

The questionnaire comprised multiple-choice, sliding-scale, or free-text answers and consisted of 4 sections (Multimedia Appendix 1). For caregivers, some questions related to the patient they cared for, whereas others related specifically to their caregiver experiences. The first section of questions collected information on the patient’s/caregiver's profiles (sex, age, spasticity diagnosis and symptoms, treatments for spasticity, duration of BoNT-A treatment, BoNT-A formulation, relationship to patient, duration and frequency of caregiving). Respondents were screened out of the survey at this stage if the eligibility criteria were not met. In countries other than Spain, patients selected the BoNT-A formulation from the following list: Dysport (aboBoNT-A), Xeomin (incobotulinumtoxinA, incoBoNT-A), Botox (onabotulinumtoxinA; onaBoNT‑A), or “not known.” Patients in Spain were asked to state the BoNT-A treatment they were receiving (if known) in a free-text field.

The second section collected information on the impact of spasticity on the ability to work, functioning, and QoL. The third section collected information on BoNT-A treatment behavior (goals, number of injections, and retreatment) and the impact of BoNT-A injections on patients' and caregivers' QoL. The final section collected information on the potential impact of reduced BoNT-A injection frequency on patients and caregivers (assuming efficacy was maintained). The sliding-scale questions in these sections are shown in Multimedia Appendix 1.

The responses provided by the patients or caregivers regarding the patients' condition were self-reported and were not verified by an independent rater or reviewer. Free-text responses were categorized both automatically and manually (as appropriate) by Carenity personnel, who also developed a tool to program and code the questionnaire.

### Eligibility Criteria

Eligible participants were adult patients (≥18 years old) who self-reported as having spasticity and receiving treatment with BoNT-A for ≥1 year, and caregivers of patients meeting the survey criteria (caregivers were not those of the participating patients). Spasticity had to be due to MS, stroke, traumatic brain injury, spinal cord injury, cerebral palsy, brain tumor, or spastic paraplegia. Patients treated with oral antispasticity medications (eg, baclofen) or receiving concurrent physiotherapy (at home or in hospital) were permitted to participate.

### Statistical Analyses

Descriptive analyses are presented: categorical variables are presented as frequency counts and percentages. Differences in reported burden, treatment behavior, and perceived benefits of fewer injections are compared between patients by limbs affected, level of difficulty experienced due to spasticity, symptoms experienced, and treatment received (number of injections).

### Compliance

The study was conducted in accordance with Good Pharmacovigilance Practices Modules IV and VIII in compliance with relevant codes of conduct and data protection legislation, with no approval from the Clinical Research Ethics Committee or Independent Review Board required. All participants provided informed consent to participate and were made aware that the research was sponsored by a pharmaceutical company that manufactures a product approved for the treatment of spasticity.

### Funding and Data Sharing

This study was sponsored by Ipsen. Where patient data can be anonymized, Ipsen will share all data that underlie the results reported in this paper with qualified researchers who provide a valid research question. Study documents, such as the study protocol and clinical study report, are not always available. Proposals should be submitted to DataSharing@Ipsen.com and will be assessed by a scientific review board. Data are available beginning 6 months and ending 5 years after publication; after this time, only raw data may be available.

## Results

### Patient Demographics and Clinical Characteristics

**Figure 1:**
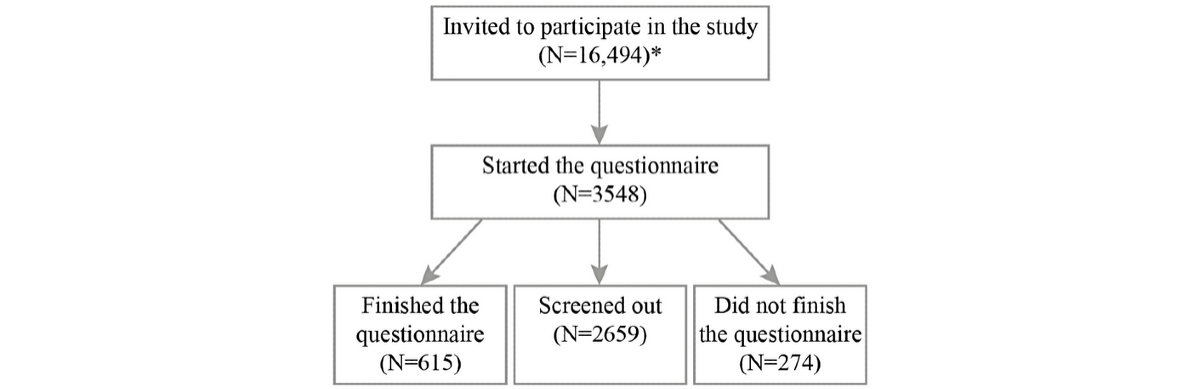
Disposition of survey respondents. *Respondents who had agreed to receive invitations to participate in questionnaires.

Carenity invited 16,494 members (who had agreed to receive invitations to participate in questionnaires) from their community of members affected by spasticity or one of the targeted diseases to complete the survey and, of these, 3548 members started the survey (Figure 1). Participants were screened out of the study if they did not meet the eligibility criteria (n=2,659), and 274 participants did not complete the questionnaire.

In total, 615 participants completed the survey (427 patients and 188 caregivers), and the survey was closed (United States, 300/615, 49%; Europe, 315/615, 51%; Table 1). Patients had a mean age of 41.7 (95% CI 40.6-42.8) years; 48% (206/427) were women, 51% (216/427) were men, and 1% (5/427) were transgender.

The mean age at diagnosis was 33.5 (95% CI 32.3-34.7) years, and the mean time since diagnosis was 8.3 (95% CI 7.4-9.3) years. More patients had spasticity due to MS (199/427, 47%) than other conditions (Table 1). The proportion of patients with MS was lower in the United States (65/178, 37%) than in Europe (134/249, 54%; Multimedia Appendix 2). The most frequent symptoms of spasticity were muscle spasms, stiffness/rigidity, and muscle pain (Table 1).

The current BoNT-A treatment was onaBoNT-A for most patients (237/427, 56%), with 18% (75/427) receiving aboBoNT-A and 11% (48/427) receiving incoBoNT-A (Table 1). The mean time since the first BoNT-A treatment was 3.5 (95% CI 3.0-3.9) years, suggesting an average gap of 4.8 years between diagnosis and BoNT-A initiation. More patients from the participating European countries did not know which formulation they were taking (21% vs 6% for the United States; Multimedia Appendix 2). In total, 60% (256/427) of patients were receiving concurrent oral medication (eg, muscle relaxants or baclofen), 69% (297/427) were receiving physiotherapy (35% at home, 34% in hospital), 11% (45/427) had received phenol, 9% (40/427) had received botulinum neurotoxin type B injections, and 7% (29/427) had received alcohol (Table 1).

**Table 1:** Participant characteristics (N=615).

Characteristic		Patients (n=427)	Caregivers (n=188)	Caregivers' patients (n=188)
**Age in years, n (%)**				
	18-30	72 (17)	49 (26)	24 (13)
	31-40	127 (30)	63 (34)	15 (8)
	41-50	134 (31)	45 (24)	25 (13)
	51-65	79 (19)	27 (14)	53 (28)
	≥66	15 (4)	4 (2)	71 (38)
**Gender, n (%)**				
	Men	216 (51)	84 (45)	84 (45)
	Women	206 (48)	104 (55)	104 (55)
	Transgender	5 (1)	0 (0)	0 (0)
**Relationship of patient to caregiver, n (%)**				
	Parent	N/A^a^	76 (40)	N/A
	Another family member	N/A	51 (27)	N/A
	Friend	N/A	17 (9)	N/A
	Partner	N/A	14 (7)	N/A
	Child	N/A	11 (6)	N/A
	Sibling	N/A	11 (6)	N/A
	Neighbor	N/A	6 (3)	N/A
	Other	N/A	2 (1)	N/A
**Frequency (days per week) of caregiving, n (%)**				
	≥1	N/A	14 (7)	N/A
	≥2	N/A	27 (14)	N/A
	≥4	N/A	62 (33)	N/A
	Daily	N/A	85 (45)	N/A
**Duration of caregiving in years, n (%)**				
	<1	N/A	11 (6)	N/A
	1-3	N/A	64 (34)	N/A
	3-5	N/A	46 (24)	N/A
	5-10	N/A	45 (24)	N/A
	>10	N/A	22 (12)	N/A
**Cause of spasticity, n (%)**				
	Brain tumor	13 (3)	N/A	5 (3)
	Cerebral palsy	31 (7)	N/A	19 (10)
	Multiple sclerosis	199 (47)	N/A	57 (30)
	Spastic paraplegia	41 (10)	N/A	20 (11)
	Spinal cord injury	40 (9)	N/A	20 (11)
	Stroke	69 (16)	N/A	53 (28)
	Traumatic brain injury	34 (8)	N/A	14 (7)
**Time (in years) since diagnosis, n (%)**				
	<3	140 (33)	N/A	44 (23)
	3-5	61 (14)	N/A	42 (22)
	5-10	79 (19)	N/A	57 (30)
	10-15	54 (13)	N/A	10 (5)
	>15	74 (17)	N/A	30 (16)
	Not specified	19 (4)	N/A	5 (3)
**Symptoms experienced, n (%)**				
	Difficulties using arm(s)	194 (45)	N/A	108 (57)
	Difficulties using legs	286 (67)	N/A	133 (71)
	Muscle pain	295 (69)	N/A	129 (69)
	Muscle spasms	308 (72)	N/A	125 (66)
	Muscle stiffness/rigidity	295 (69)	N/A	136 (72)
	Unwanted movement of the stiff limb	176 (41)	N/A	64 (34)
**Botulinum neurotoxin type A treatment received,^b^ n (%)**				
	AbobotulinumtoxinA	75 (18)	N/A	34 (18)
	IncobotulinumtoxinA	48 (11)	N/A	19 (10)
	OnabotulinumtoxinA	237 (56)	N/A	101 (54)
	Other^c^	3 (0)	N/A	0 (0)
	Do not know	64 (15)	N/A	34 (18)
**Time (in years) since treatment initiation, n (%)**				
	<2	203 (48)	N/A	68 (36)
	2-5	118 (28)	N/A	68 (36)
	5-10	61 (14)	N/A	40 (21)
	10-15	29 (7)	N/A	8 (4)
	>15	16 (4)	N/A	4 (2)
Mean time since treatment initiation, years (95% CI)		3.5 (3.0-3.9)	N/A	3.5 (2.9-4.1)
**Concomitant therapy, n (%)**				
	Alcohol injections	29 (7)	N/A	8 (4)
	Botulinum B injections	40 (9)	N/A	24 (13)
	Intrathecal baclofen injections	37 (9)	N/A	17 (9)
	Phenol injections	45 (11)	N/A	13 (7)
	Oral medications^d^	256 (60)	N/A	109 (58)
	Physiotherapy at home	151 (35)	N/A	78 (41)
	Physiotherapy at hospital/clinic	146 (34)	N/A	56 (30)
	Self-rehabilitation (home-based)	79 (19)	N/A	65 (35)
	Other	2 (0)	N/A	0 (0)

^a^N/A: Not applicable.

^b^Self-reported.

^c^For respondents from Spain only, brand names given were Bocouture, Lantox, and Azzalure.

^d^Eg, muscle relaxants, baclofen.

### Caregiver Profiles

The mean age of caregivers was 38.6 (95% CI 36.9-40.2) years; 55% (104/188) were women and 45% (84/188) were men (Table 1). The mean duration of caregiving was reported as 4.9 (95% CI 4.1-5.7) years, with 12% (22/188) providing care for ≥10 years. Most caregivers (147/188, 78%) provided care either every day (85/188, 45%) or for ≥4 days a week (62/188, 33%). The patient was a parent of the caregiver in 40% (76/188) of cases, with the remainder being another family member, a friend, a partner, a neighbor, or other. The mean age of caregivers was highest in those providing care for patients with stroke (41.7 years; 95% CI 38.7-44.7) and lowest for traumatic brain injury (35.2 years; 95% CI 30.2-40.2). For MS—the most common cause of spasticity in this study—caregivers had a mean age of 37.6 (95% CI 34.7-40.6) years.

Caregiver profiles were similar in Europe and the United States, although the latter appeared to spend more time each week in their caregiving role (Multimedia Appendix 2).

### Burden of Spasticity

#### Employment

Overall, of the 427 patients, 412 patients (96%) were aged <65 years; 69% (284/412) were employed, 1% (6/412) were full-time students, and 30% (122/412) were unemployed (Table 2). Among patients aged <65 years, 44% (181/412) reported that their condition had an impact on their professional status, 22% (91/412) reported having to work part-time, and 22% (90/412) were unable to work (Table 2).

Most caregivers (184/188, 98%) were also aged <65 years. Of these, 29% (53/184) reported that caring for their patient had an impact on their own professional status, including 21% (38/184) who reported having to take a part-time job and 8% (15/184) who did not work in order to take care of their patient (Table 2).

Compared with the participating European countries, more caregivers in the United States changed their working situation to care for the patient (Table 3).

**Table 2:** Burden of spasticity and botulinum neurotoxin type A (BoNT-A) treatment, and the perceived benefits of fewer BoNT-A treatments, for patients and caregivers (N=615).

Variables		Patients (N=427)	Caregivers (N=188)
**Employment^a^, n (%)**		n=412	n=184
	Full-time	175 (43)	98 (53)
	Part-time, due to condition/caregiving	91 (22)	38 (21)
	Do not work due to condition/caregiving	90 (22)	15 (8)
	Part-time, not due to condition/caregiving	18 (4)	16 (9)
	Do not work, not due to condition/caregiving	32 (8)	8 (4)
	Full-time student	6 (1)	7 (4)
	Other	0 (0)	2 (1)
**Impact of spasticity on time spent at work^a^, n (%)**		n=412	n=184
	Impact	181 (44)	53 (29)
	No impact	231 (56)	129 (70)
	Other	0 (0)	2 (1)
**Time off work due to BoNT-A injection^b^, n (%)**		n=285	n=156
	Never	64 (22)	25 (16)
	Sometimes	149 (52)	96 (62)
	Often	39 (14)	25 (16)
	Always	33 (12)	10 (6)
**Number of days taken off work per year due to BoNT-A injection^b^, n (%)**		n=216	n=121
	≤2	51 (24)	21 (17)
	3-4	52 (24)	38 (31)
	5-9	64 (30)	26 (22)
	10-15	26 (12)	18 (15)
	>15	23 (11)	18 (15)
**Cost (€^c^) per treatment, n (%)**		n=330	n=153
	0-10	27 (8)	15 (10)
	10-30	47 (14)	30 (20)
	30-50	27 (8)	14 (9)
	50-100	41 (12)	35 (23)
	100-300	84 (26)	43 (28)
	>300	104 (32)	16 (10)
**Out-of-pocket costs^d^, n (%)**		n=427	n=188
	Consultations	153 (36)	61 (32)
	Parking	183 (43)	82 (44)
	Transportation	235 (55)	114 (61)
	Treatments	165 (39)	50 (27)
	Reduced salary^b^	99 (23)	63 (34)
	Other^e^	2 (0)	0 (0)
	None	83 (19)	32 (17)
**3 most important perceived benefits of fewer treatments, n (%)**		n=427	n=188
	Longer periods with improved mobility	196 (46)	N/A^f^
	Longer periods not worrying about symptoms	169 (40)	124 (66)
	More self-confidence	120 (28)	N/A
	Less impact on work activities	115 (27)	92 (49)
	More quality time with family and friends	110 (26)	103 (55)
	Higher self-esteem	106 (25)	N/A
	Less dependence on others	94 (22)	N/A
	Less logistical burden	117 (27)	100 (53)
	Reliving my fear of injections less frequently	79 (19)	N/A
	Less financial burden	1 (0)	2 (1)
	Improved quality of life	N/A	1 (1)
	I would not experience any benefits	14 (3)	6 (3)

^a^Only including participants who are younger than 65 years of age.

^b^Among those who worked part-time or full-time or answered “other” (patients, n=285; caregivers, n=156); the corresponding percentages are 35% and 40%, respectively.

^c^A currency exchange rate of eur 1€=US $1.18 is applicable.

^d^Only including patients (n=330) and caregivers (n=153) who have to pay something for BoNT-A injections (excluding 14 patients and 3 caregivers who did not answer).

^e^Food for patient or driver.

^f^N/A: not applicable.

**Table 3:** Burden of spasticity and botulinum neurotoxin type A (BoNT-A) treatment, and the perceived benefits of fewer BoNT-A treatments, for patients and caregivers in Europe and the United States (N=615).

Variables		Patients & caregivers in Europe (N=315)		Patients & caregivers in the United States (N=300)	
		Patients	Caregivers	Patients	Caregivers
**Employment^a^, n (%)**		n=239	n=65	n=173	n=119
	Full-time	92 (38)	38 (58)	83 (48)	60 (50)
	Part-time, due to condition/caregiving	48 (20)	10 (15)	43 (25)	28 (24)
	Do not work, due to condition/caregiving	60 (25)	3 (5)	30 (17)	12 (10)
	Part-time, not due to condition/caregiving	10 (4)	7 (11)	8 (5)	9 (8)
	Do not work, not due to condition/caregiving	27 (11)	0 (0)	5 (3)	8 (7)
	Full-time student	2 (1)	6 (9)	4 (2)	1 (1)
	Other	0 (0)	1 (2)	0 (0)	1 (1)
**Impact of spasticity on time spent at work^a^, n (%)**		n=239	n=65	n=173	n=119
	Impact	108 (45)	13 (20)	73 (42)	40 (34)
	No impact	131 (55)	51 (78)	100 (58)	78 (66)
	Other	0 (0)	1 (2)	0 (0)	1 (1)
**Time off work due to BoNT-A injection^b^, n (%)**		n=150	n=57	n=135	n=99
	Never	38 (25)	7 (12)	26 (19)	18 (18)
	Sometimes	70 (47)	39 (68)	79 (59)	57 (58)
	Often	18 (12)	9 (16)	21 (16)	16 (16)
	Always	24 (16)	2 (4)	9 (7)	8 (8)
**Number of days taken off work per year due to BoNT-A injection^b^, n (%)**		n=109	n=47	n=107	n=81
	≤2	29 (27)	11 (23)	22 (21)	10 (12)
	3-4	25 (23)	9 (19)	27 (25)	29 (36)
	5-9	30 (28)	9 (19)	34 (32)	17 (21)
	10-15	12 (11)	11 (23)	14 (13)	7 (9)
	>15	13 (12)	7 (15)	10 (9)	18 (22)
**Cost (€^c^) per treatment, n (%)**		n=180	n=54	n=150	n=99
	0-10	20 (11)	9 (17)	7 (5)	6 (6)
	10-30	30 (17)	10 (19)	17 (11)	20 (20)
	30-50	14 (8)	5 (9)	13 (9)	9 (9)
	50-100	18 (10)	13 (24)	23 (15)	22 (22)
	100-300	38 (21)	11 (20)	46 (31)	32 (32)
	>300	60 (33)	6 (11)	44 (29)	10 (10)
**Out-of-pocket costs^d^, n (%)**		n=249	n=66	n=178	n=122
	Consultations	64 (26)	17 (26)	89 (50)	44 (36)
	Parking	105 (24)	34 (52)	78 (44)	48 (39)
	Transportation	130 (52)	41 (62)	105 (59)	73 (60)
	Treatments	66 (27)	15 (23)	99 (56)	35 (29)
	Reduced salary^b^	34 (14)	21 (32)	65 (37)	42 (34)
	Other^e^	0 (0)	0 (0)	2 (1)	0 (0)
	None	62 (25)	11 (17)	21 (12)	21 (17)
**3 most important perceived benefits of fewer treatments, n (%)**		n=249	n=66	n=178	n=122
	Longer periods with improved mobility	118 (47)	N/A^f^	78 (44)	N/A
	Longer periods not worrying about symptoms	94 (38)	41 (62)	75 (42)	83 (68)
	More self-confidence	68 (27)	N/A	52 (29)	N/A
	Less impact on work activities	60 (24)	30 (45)	55 (31)	62 (51)
	More quality time with family and friends	51 (20)	32 (48)	59 (33)	71 (58)
	Higher self-esteem	53 (21)	N/A	53 (30)	N/A
	Less dependence on others	48 (19)	N/A	46 (26)	N/A
	Less logistical burden	75 (30)	31 (47)	42 (24)	69 (57)
	Reliving my fear of injections less frequently	50 (20)	N/A	29 (16)	N/A
	Less financial burden	0 (0)	0 (0)	1 (1)	2 (2)
	Improved quality of life	N/A	0 (0)	N/A	1 (1)
	I would not experience any benefits	11 (4)	2 (3)	3 (2)	4 (3)

^a^Only including participants who are younger than 65 years of age.

^b^Of 441 participants (patients, n=285; caregivers, n=156) who worked part-time or full-time or answered “other.”

^c^A currency exchange rate of eur 1€=US $1.18 is applicable.

^d^Only including patients (n=330) and caregivers (n=153) who have to pay something for BoNT-A injections (excluding 14 patients and 3 caregivers who did not answer).

^e^Food for patient and/or driver.

^f^N/A: not applicable.

#### Daily Living and Quality of Life

**Figure 2:**
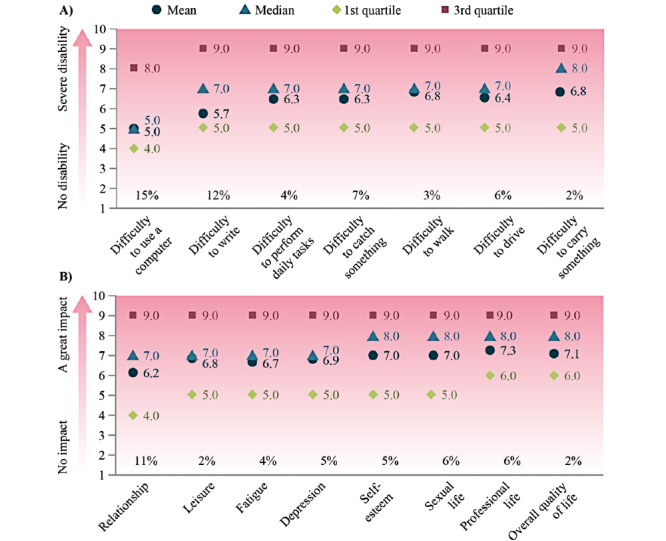
Patient responses on the impact of spasticity on the patients’ (A) ability to perform everyday tasks (“For each of the following items, please assess the level of difficulty you experience due to your spasticity,” on a scale of 0=no disability to 10=severe disability) and (B) quality of life (“Please assess to what extent spasticity affects your life,” on a scale of 0=no impact to 10=a great impact; n=427).

Scores for the difficulties associated with daily living because of spasticity, as reported by patients, are shown in Figure 2. All median scores were ≥5.0, and the task most affected was carrying things.

At least 85% (364/427) of patients experienced difficulties in ≥1 aspect of daily living; aspects most frequently affected were the ability to carry something (418/427, 98%), walking (414/427, 97%), performing daily tasks (410/427, 96%), and driving (403/427, 94%). Patients with >2 affected limbs experienced the most difficulties with daily living, with 91% (114/125) of patients experiencing 7 difficulties. Patients whose lower limbs only were affected experienced the fewest difficulties, with only 47% (39/83) of patients experiencing 7 difficulties.

Scores for the impact of spasticity on patients' QoL are shown in Figure 2. All median scores were ≥7.0, with professional life, overall QoL, sexual life, and self-esteem being most affected. At least 89% (381/427) of patients reported that spasticity affected ≥1 aspect of their QoL, with leisure (419/427, 98%) and overall QoL (420/427, 98%) being most affected.

**Figure 3:**
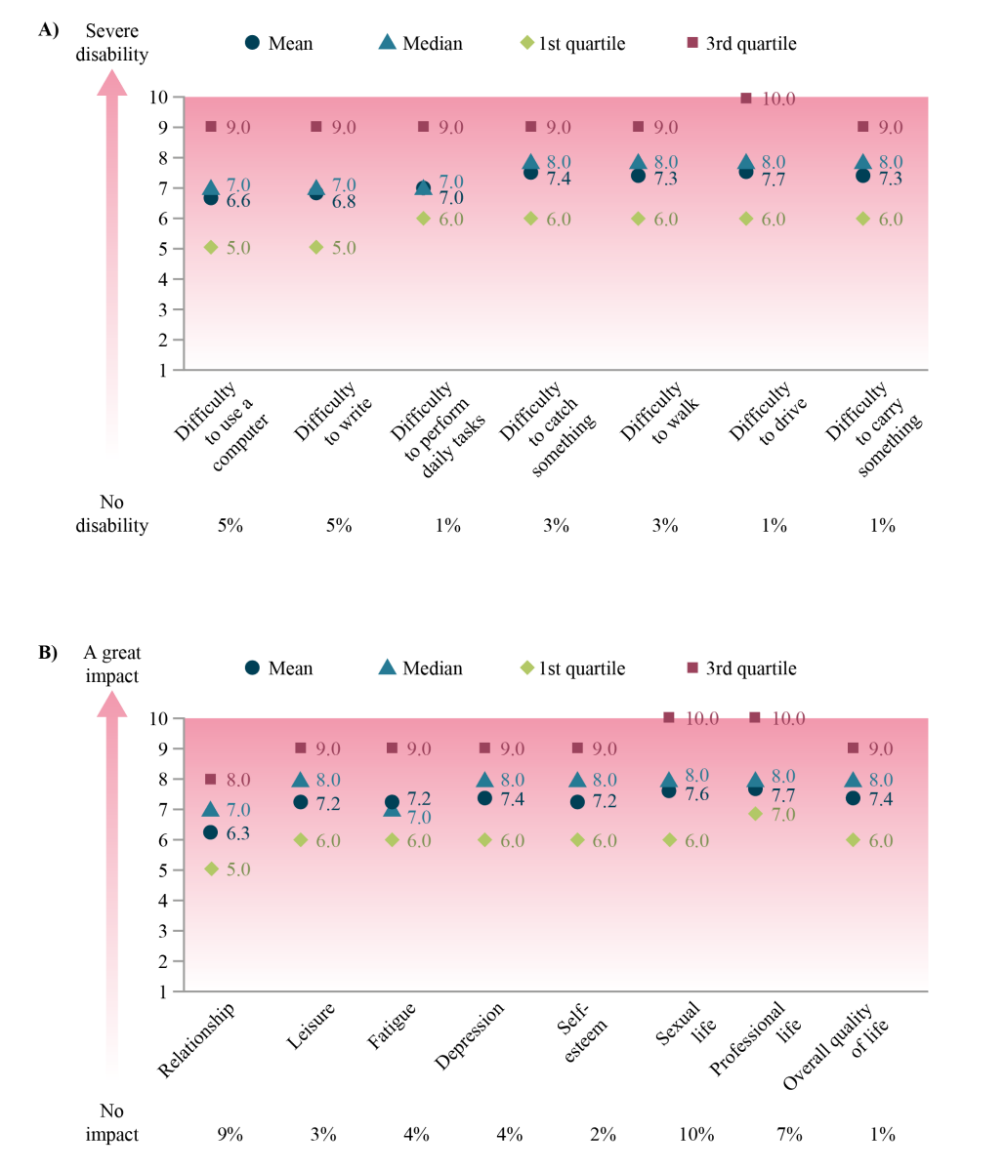
Caregiver responses on the impact of spasticity on the patients’ (A) daily life ("For each of the following items, please assess the level of difficulty you experience due to your spasticity," on a scale of 0=no disability to 10=severe disability) and (B) quality of life ("Please assess to what extent spasticity affects your life," on a scale of 0=no difficulty to 10=great difficulty; n=188).

Scores for impact on daily living and QoL reported by caregivers (on behalf of patients) are summarized in Figure 3. Caregivers gave slightly higher scores than patients for difficulties with daily living.

#### BoNT-A Treatment Behavior

**Figure 4:**
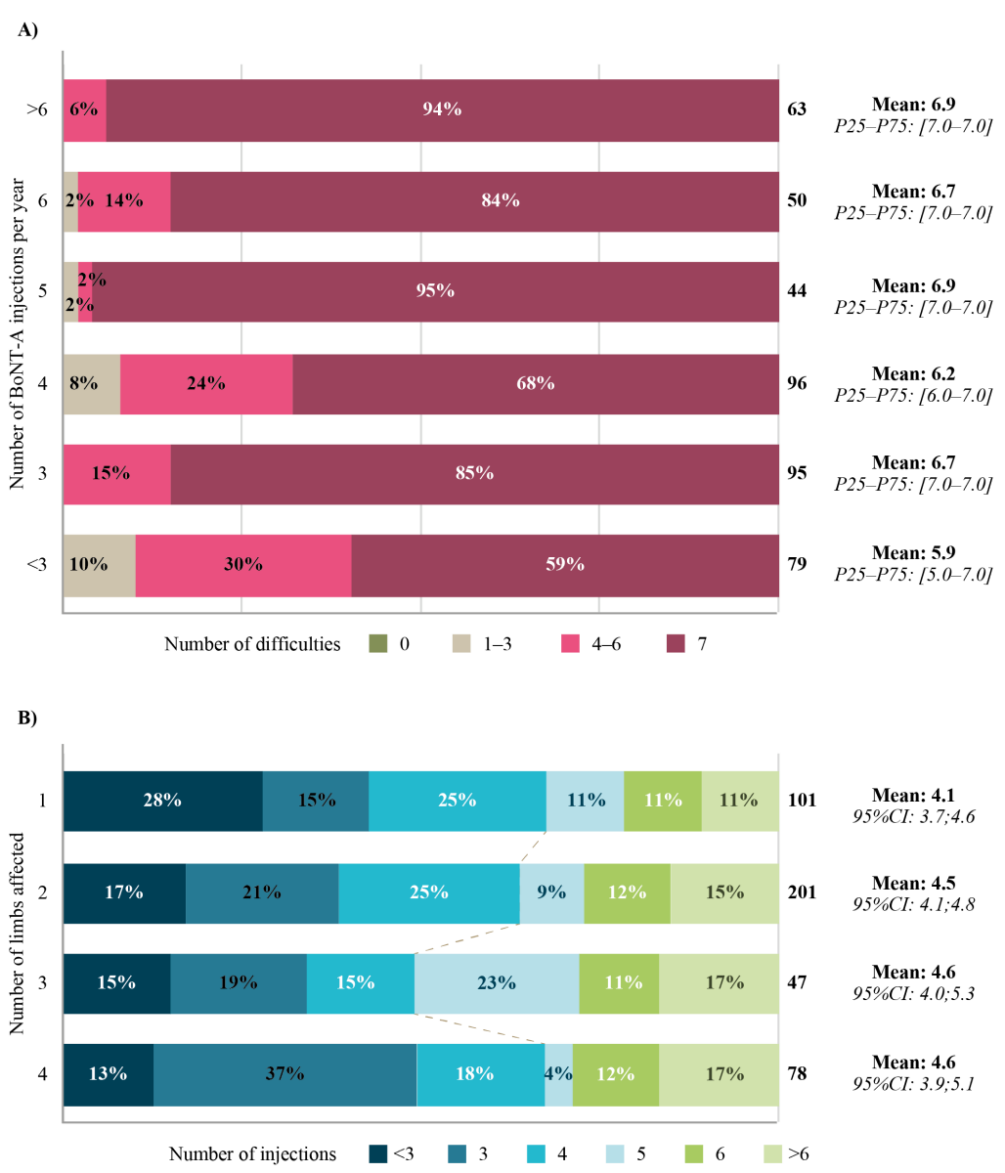
Patient responses to (A) the number of difficulties in activities of daily living, according to the number of botulinum neurotoxim type A (BoNT-A) injections received each year ["On average, how many BoNT-A treatments do you receive per year (numeric response)?" "For each of the following items, please assess the level of difficulty you experience due to your spasticity" (scale, 0=no difficulty and 10=severe difficulty)] and (B) the number of limbs affected according to the number of BoNT-A injections received each year ["In which (how many) limbs do you experience spasticity symptoms (multiple-choice response)?" "On average, how many BoNT-A treatments do you receive per year (numeric response)"](n=427). P = percentile.

The median (25^th^ percentile-75^th^ percentile) number of patient-reported injections per year was 4.0 (3.0-6.0; 26% of patients received ≥6 injections per year) and was consistent across primary conditions, ranging from 4.0 (3.0-4.0, cerebral palsy) to 5.0 (4.0-8.0, traumatic brain injury), with the exception of brain tumors, which had very few respondents (13/427) and a median (25^th^ percentile-75^th^ percentile) number of reported injections per year of 3.0 (2.0-3.0). Patients receiving <3 injections per year reported fewer difficulties, and the number of injections per year was not related to the number of affected limbs (Figure 4). The median (25^th^ percentile-75^th^ percentile) number of injections per year was the same between formulations of BoNT-A (4.0, 3.0-6.0, for onaBoNT‑A, incoBoNT-A, and aboBoNT-A).

**Figure 5:**
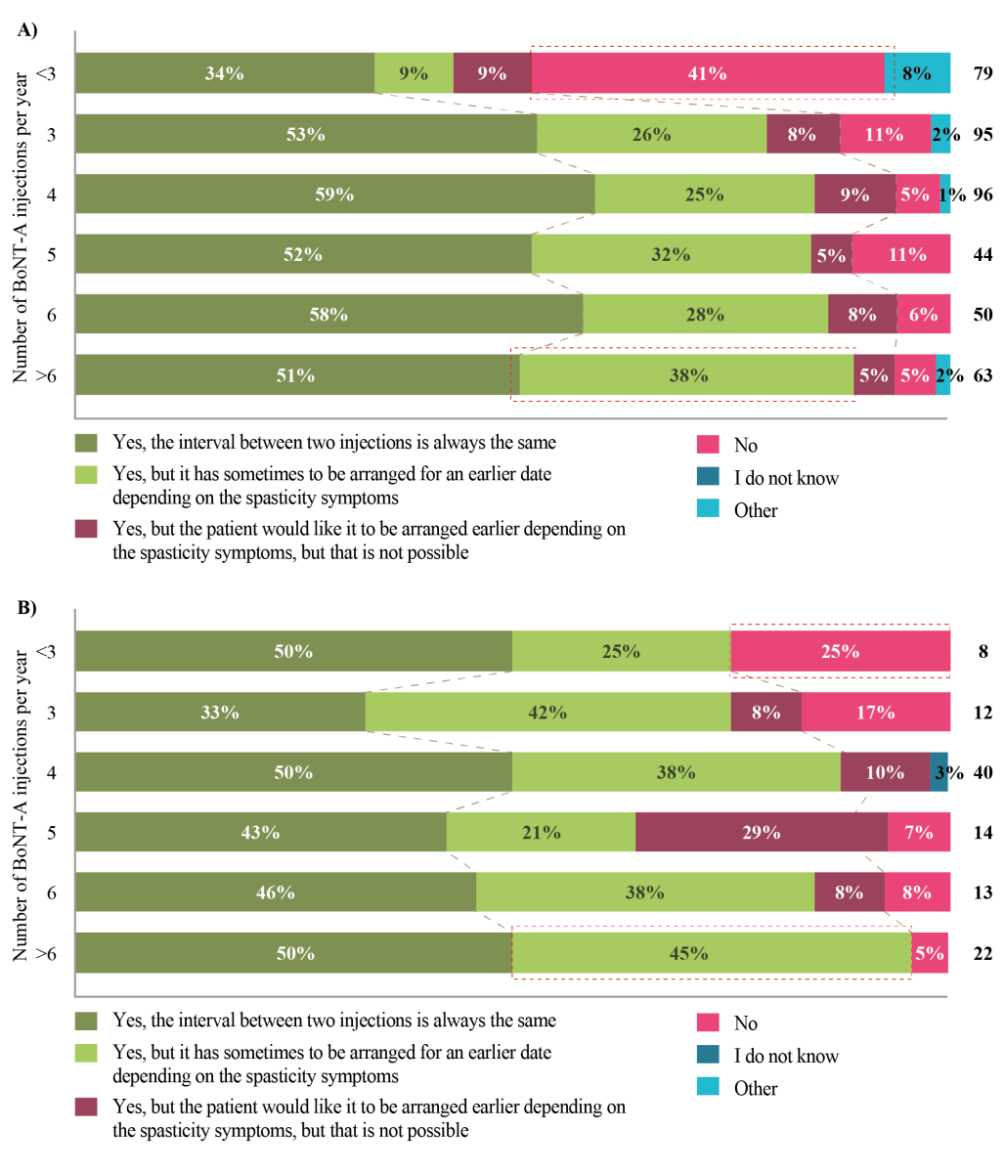
How A) patients (n=427) and B) caregivers (n=109) plan the next treatment date by the number of botulinum neurotoxin A (BoNT-A) treatments received per year ("Do you plan the next treatment date immediately after you get your injections of BoNT-A with your doctor (single-choice response)?" "On average, how many BoNT-A treatments do you receive per year (numeric response)?").

Most patients (371/427, 87%) and caregivers (151/188, 80%) reported that treatment goals were discussed with doctors. The majority of patients (359/427, 84%) also indicated that retreatment was planned immediately after a treatment. This depended on the number of injections per year; retreatment was *not* planned in 41% (32/79) of patients receiving <3 injections per year, compared with 11% (10/95) of patients receiving 3 injections per year, 5% (5/96) of patients receiving 4 injections per year, 11% (5/44) of patients receiving 5 injections per year, 6% (3/50) of patients receiving 6 injections per year, and 5% (3/63) of patients receiving >6 injections per year. In approximately one-third of cases (108/359), earlier retreatment sometimes had to be arranged due to spasticity symptoms, and in 9% (33/359) of cases, patients would have liked to have had earlier retreatment than scheduled, but this was not possible. Among those patients who received >6 injections per year, 38% (24/63) reported that they had to receive earlier retreatment due to spasticity symptoms, whereas for those patients who received <3 injections per year, only 9% (7/79) reported that they had to receive earlier retreatment due to spasticity symptoms (Figure 5). Caregiver responses for retreatment planning according to the number of BoNT-A treatments per year are presented in Figure 5.

### Burden of Receiving BoNT-A Injections

#### Employment

The majority of employed patients (221/285, 78%) and caregivers (131/156, 84%) had to take time off from employment (defined as any part of a working day) for BoNT-A treatment, and >50% of patients and caregivers took ≥5 days off per year (Table 2).

As expected, the number of days patients took off work to receive injections appeared to increase with the number of injections per year. Of the 32 patients who received <3 injections per year, 38% (12/32) required ≥3 days off work, compared with 73% (24/33) of patients who received 3 injections per year, 74% (32/43) of patients who received 4 injections per year, 90% (27/30) of patients who received 5 injections per year, 85% (29/34) of patients who received 6 injections per year, and 93% (41/44) of patients who received >6 injections per year.

#### Issues Associated With BoNT-A Treatment

At least 73% (312/427) of patients reported issues with BoNT-A treatment; cost, availability of timely appointments, fear of injections, and frequency of injections represented the greatest issues [median (25^th^ percentile-75^th^ percentile) scores of 7.0 (4.0-9.0), 7.0 (4.0-8.0), 6.0 (3.0-8.0), and 6.0 (4.0-8.0), respectively]. Among caregivers, at least 84% (157/188) reported issues with BoNT-A treatment, with the cost of injections and logistics representing the greatest burdens to them [median (25^th^ percentile-75^th^ percentile) score 6.0 (5.0-8.0) and 6.0 (4.0-8.0), respectively].

#### Cost

Overall, 77% of patients (330/427) and 81% of caregivers (153/188) reported that they incurred costs at the time of each BoNT-A injection. Most patients (303/330, 92%) and caregivers (138/153, 90%) bore a financial cost of >10€ with each BoNT-A treatment, with 57% (188/330) of patients and 39% (59/153) of caregivers paying >100€ with each BoNT-A treatment (Table 2). Over 1 year, 53% (176/330) of patients reported spending >500€ to receive BoNT-A treatment, and the mean cost per year was 1080€ (95% CI 923-1236). (A currency exchange rate of eur 1€=US $1.18 is applicable.)

Out-of-pocket expenses were experienced by most patients (344/427, 81%) and caregivers (156/188, 83%), with transportation costs and parking fees the most common (Table 2). Among working participants, 35% (99/285) of patients and 40% (63/156) of caregivers reported a reduced salary due to time off work for BoNT-A treatment. More patients in the United States than in participating European countries reported out-of-pocket expenses (Table 3).

### Benefits and Concerns About BoNT-A Treatment

#### Improvements Due to BoNT-A Treatment

Overall satisfaction with life improved with BoNT-A treatment for 92% (393/427) of patients, with a median (25^th^ percentile-75^th^ percentile) improvement score of 7.0 (5.0-9.0). For individual daily task and QoL domains, improvements were reported for 81%-94% (345/427-402/427) of patients, with a median (25^th^ percentile-75^th^ percentile) score for all tasks and domains of 7.0 (5.0-8.0).

Scores for improvements due to BoNT-A reported by patients and caregivers (on behalf of patients) were similar and are presented in Table 4.

**Table 4:** Scores for patients’ and caregivers’ responses on improvements in patients’ lives with botulinum neurotoxin type A (BoNT‑A) treatment; improvement was rated on a scale of 0-10, where 0=no improvement and 10=greatly improved (n=615).

Daily task and QoL^a^ domains	Patient (n=427) scores, on a scale of 0-10				Caregiver (n=188) scores, on a scale of 0-10			
	Mean	Median	25^th^ percentile	75^th^ percentile	Mean	Median	25^th^ percentile	75^th^ percentile
Muscle spasm	6.7	7.0	5.0	8.0	6.6	7.0	6.0	8.0
Pain	6.5	7.0	5.0	8.0	6.4	6.0	5.0	8.0
Ability to perform daily tasks	6.6	7.0	5.0	8.0	6.3	7.0	5.0	8.0
Ability to walk	6.6	7.0	5.0	8.0	6.3	6.0	5.0	8.0
Transfers (moving around, short trips)	6.5	7.0	5.0	8.0	6.3	6.0	5.0	8.0
Fatigue	6.5	7.0	5.0	9.0	6.2	6.0	5.0	8.0
Self-confidence	6.5	7.0	5.0	8.0	6.4	7.0	5.0	8.0
Leisure	6.5	7.0	5.0	8.0	6.3	7.0	5.0	8.0
Personal relationships	6.7	7.0	5.0	9.0	6.5	7.0	5.0	8.0
Ability to socialize	6.6	7.0	5.0	8.0	6.5	7.0	5.0	8.0
Willingness to perform activities	6.7	7.0	5.0	9.0	6.7	7.0	5.0	8.0
Depression	6.5	7.0	5.0	8.0	6.2	6.0	5.0	8.0
Professional life	6.7	7.0	5.0	9.0	6.1	6.0	5.0	8.0
Sexual life	6.5	7.0	5.0	8.0	5.9	6.0	4.0	8.0
Anxiety	6.4	7.0	5.0	8.0	6.1	6.0	5.0	8.0
Overall satisfaction	6.7	7.0	5.0	9.0	6.5	7.0	5.0	8.0

^a^QoL: quality of life.

#### Issues or Concerns About BoNT-A Injections

In response to an open question exploring the main issues or concerns with treatment, patients’ most frequently reported issue/concern was side effects (171/427, 40%), namely long‑term risks (52/427, 12%), muscular issues (18/427, 4%), urinary incontinence (5/427, 1%), and infections (3/427, 0.7%). Other common concerns included issues with treatment efficacy (95/427, 22%) and administration (75/427, 18%). The former included concerns about lack of effectiveness (51/427, 12%) and long-term loss of efficacy (37/427, 9%), whereas the latter included concerns about pain (27/427, 6%), cost (14/427, 3%), and method of administration (10/427, 2%). In response to the same question, the most frequently reported issue/concern from caregivers was also side effects (93/188, 49%), in particular long-term risks (36/188, 19%). Caregivers also reported concerns regarding the efficacy of treatment (55/188, 29%), namely lack of effectiveness (27/188, 14%), administration of treatment (25/188, 13%), and concerns regarding dosage (6/188, 3%).

No issues or concerns were reported by 15% (63/427) of patients and 10% (18/188) of caregivers.

#### Perceived Benefits of Requiring Fewer BoNT-A Injections

Assuming a longer duration of effect of BoNT-A, most patients (368/427, 86%) and caregivers (163/188, 87%) believed that they would see fewer BoNT-A treatments per year as a benefit.

In response to an open question posed to 368 patients who believed that fewer BoNT-A injections would be beneficial), the most frequently cited perceived benefits were improved QoL (63/368, 17%), fewer logistical constraints (60/368, 16%), and improved psychological wellbeing (56/368, 15%). At least 79% (338/427) of all patients reported that fewer BoNT-A treatments would improve ≥1 aspect of treatment burden, with a median (25^th^ percentile-75^th^ percentile) improvement score of 8.0 each for logistics (5.5-9.0), cost of getting injections (6.0-9.0), and general impact on QoL (6.0-9.0). In response to a prespecified list of potential benefits, the 3 most important benefits of fewer injections reported were longer periods of improved mobility (196/427, 46%), not worrying about symptoms (169/427, 40%), and more self-confidence (120/427, 28%; Table 2).

In response to an open question to 163 caregivers who believed that fewer BoNT-A injections for their patients would be beneficial to themselves, the most commonly anticipated benefits were fewer logistical constraints (37/163, 23%), lower out-of-pocket expenses (33/163, 20%), and improved psychological wellbeing (30/163, 18%). The 3 most important reported perceived benefits of fewer injections for all caregivers were longer periods not worrying about symptoms (124/188, 66%), more quality time with family and friends (103/188, 55%), and less logistical burden (100/188, 53%; Table 2).

Only 3% of patients (14/427) and caregivers (6/188) reported not expecting to see any benefits with fewer injections (Table 2).

For patients, the more BoNT-A injections per year they received, the more benefit they associated with requiring fewer injections: 65% (51/79) who received <3 injections per year expected to feel some or many benefits, compared with 71%-88% receiving ≥3 injections per year [ranging from 71% (67/95) of patients who received 3 injections per year to 88% (44/50) of patients who received 6 injections per year]. Patients who reported experiencing difficulties in receiving BoNT-A treatment were more likely to expect benefits with less frequent injections: depending on the difficulties experienced, 74%-86% (273/427–318/427) answered “yes” to expecting benefits versus 59%-66% (35/427–39/427) who answered “no.”

#### Expected Reduction in Number of BoNT-A Injections to Achieve Perceived Benefits

The number of BoNT-A injections per year that would be required to achieve perceived benefits was 2 or 3 for most patients and caregivers [62% (228/367) and 71% (73/102), respectively]. Most patients and caregivers felt that 1 or 2 fewer injections per year would beneficially impact their lives (58% [215/367] and 60% [61/102], respectively). Patients receiving the highest number of injections reported the biggest reduction in the number of injections required to perceive benefits (Table 5). Caregiver-reported data (on behalf of their patients) are also presented in Table 5.

**Table 5:** Expected reduction in the number of botulinum neurotoxin type A (BoNT-A) injections per year to achieve perceived benefits for patients and caregivers, according to the number of injections currently being received ("Assuming the effect of botulinum toxin A injections could last longer, with how many injections per year would you feel the benefit of less frequent injections?").

Patient/caregiver & number of injections per year currently being received		Number of fewer BoNT-A injections per year needed to perceive benefits					
		0	1	2	3	4	>4
**Patients (n=367), n (%)**							
	<4 injections/yr (n=131)	42 (32)	76 (58)	9 (7)	4 (3)	0 (0)	0 (0)
	4 injections/yr (n=87)	5 (6)	34 (39)	42 (48)	4 (5)	2 (2)	0 (0)
	>4 injections/yr (n=149)	8 (5)	16 (11)	38 (25)	41 (28)	16 (11)	30 (20)
**Caregivers (n=102), n (%)**							
	<4 injections/yr (n=17)	4 (23)	11 (65)	2 (12)	0 (0)	0 (0)	0 (0)
	4 injections/yr (n=40)	0 (0)	13 (32)	24 (60)	2 (5)	1 (3)	0 (0)
	>4 injections/yr (n=45)	1 (2)	1 (2)	10 (22)	11 (25)	7 (16)	15 (33)

## Discussion

### Principal Findings

Evidence-based medicine for the treatment of spasticity requires the patient perspective on the burdens of symptoms and receiving treatment, for which there is a knowledge gap. The present survey, which was self-reported by over 600 patients and caregivers in the international Carenity online community, is, to our knowledge, the largest of its kind for those affected by spasticity. The results presented here supplement efficacy and safety data from clinical trials of BoNT-A treatment for spasticity with information on treatment benefits to patients, the effects of symptoms on everyday life, and practical issues associated with treatment. Such insights can inform the development of new patient-reported outcome measures and help guide more effective management of spasticity in clinical practice, thus informing the development of new therapeutics.

The use of online communities is becoming increasingly popular, as they can encourage patients to educate themselves about their condition, motivate patients to participate in clinical research, and are easily accessible; FDA guidance for collecting information from patients who are members of these communities is now available [1,2]. Online communities also offer methodological advantages by providing quick access to a specific patient population, supporting hypothesis generation, improving sociodemographic representativeness, and providing insights into the feasibility of a study and recruitment for future studies [2]. In this study, only one-fifth of people who had agreed to receive Carenity survey invitations actually participated in our survey, and information regarding the eligibility of those who did not participate is not available, highlighting the challenge of patient and caregiver engagement when using online platforms. However, a high response rate was still achievable as a result of the very large size of the online international community involved.

The presented results confirm that spasticity, irrespective of etiology, has a clear and negative effect on patients' and caregivers' lives. Specifically, patients reported the impact of spasticity on the ability to conduct routine, everyday activities like walking and driving, and on various aspects of QoL. Many patients also reported that spasticity affected their ability to work; among those aged <65 years, 22% changed to part-time working and 22% gave up work completely. In caregivers of working age, the corresponding values were 21% and 8%, respectively. These results are consistent with data from several studies showing that spasticity has a substantial impact on patients' activities of daily living, QoL, and independent living [14,33-35]. They also confirm the results of a study evaluating work restrictions faced by caregivers of patients with spasticity [36].

All the surveyed patients had received treatment with BoNT-A for ≥1 year. Patients and caregivers reported that treatment improved the ability to conduct daily tasks and aspects of QoL that are affected by spasticity, increasing patients' overall satisfaction with life. Despite its well-established efficacy for spasticity, there was an average gap of 4.8 years between diagnosis and BoNT-A treatment initiation in the population surveyed. However, for conditions such as MS, spasticity may not be present at the initial diagnosis, appearing only later in the disease course. In a previous internet survey among patients with spasticity, almost 50% had to wait 1 year before BoNT was started, and 23% waited >3 years. The authors propose that this may reflect the cost of treatment to health care providers or patients, or a lack of experienced injectors [14]. Regarding costs, reimbursement for BoNT-A by health insurance providers has been decreasing year after year in many countries [37]. In our survey, most patients and caregivers incurred costs at each BoNT-A injection, with >50% of patients reporting an annual expenditure of >500€.

Many patients reported practical issues with BoNT-A treatment, including the availability of timely appointments and the frequency of injections. On average, patients received 4.4 injections per year, irrespective of BoNT-A formulation, and, although most planned their visits, some reported scheduling earlier appointments for emergent spasticity symptoms. It is important to note that this was an international study, and treatment intervals were largely dependent on each participating country's health care insurance authorization; therefore, the number of injections per year may be more reflective of this rather than the duration of efficacy or physician guidance. Additionally, these results are dependent on the participant being able to accurately remember how many injections they received per year, and therefore, may be affected by memory bias.

When first asked whether reducing BoNT-A injection frequency (without reducing efficacy) would provide any benefits, 86% (368/427) of patients and 87% (163/188) of caregivers reported that it would, citing factors such as better QoL and fewer logistical constraints. Participants were later asked the same question alongside a prespecified list, from which they had to select the 3 most important benefits of less frequent injections. Only 3% (13/427) of patients and 3% (6/188) of caregivers responded that they would not experience benefits, which suggests that providing a list of potential benefits influenced responses, most likely by enabling participants to envisage benefits they had not been able to foresee previously. Importantly, the 3 most selected benefits (longer periods of improved mobility, not worrying about symptoms, and self-confidence) were related to prolonged symptom relief rather than a reduced number of injections.

### Injection Frequency

These data collectively suggest that the option of a longer-acting BoNT-A formulation would be favorably received by patients and their caregivers, to provide sustained relief from spasticity symptoms and to overcome practical issues associated with more frequent treatment, as suggested in another patient survey [38]. A recent clinical review indicates that aboBoNT-A treatment may allow long intervals between injections, suggesting long-term symptom relief [39]*.* These data appear to be corroborated by real-world evidence [40] and may be explained by recent preclinical research showing that aboBoNT-A contains more active neurotoxin at licensed doses than other BoNT products [41].

The ability to reduce injection frequency depends on the duration of the effect of the BoNT-A injection. This, in turn, depends on several factors, including dose, muscle mass, and depth of injection [42]. In a model designed to assess the pharmacodynamic effects of aboBoNT-A and onaBoNT‑A, the former had a significantly longer duration of action [43]. A Phase 3 trial of aboBoNT-A in patients with lower limb spasticity post-stroke or post-traumatic brain injury permitted retreatment (per the investigator's judgment) at Weeks 12, 16, 20, or 24 [44]. The percentages of patients re-injected at week 16 or later were 20% during the first cycle, 32% during the second cycle, and 15% during the third cycle, indicating that some patients required ≤3 aboBoNT‑A injections per year. The authors concluded that the long duration of aboBoNT-A may reduce the burden associated with injection frequency [44], which is consistent with the anticipated benefits of less frequent administration in the current study.

### Limitations

This study has several limitations. No formal prespecified statistical evaluation took place, and recruitment was conducted via the Carenity website, meaning results may not be representative of the general population of patients with spasticity and their caregivers. The proportion of patients with MS in this survey was higher than would be expected in the general population; in the United States, for example, the incidence of MS is surpassed by both stroke and traumatic brain injury [45]. This survey system self-selected for engaged participants who were familiar with social media and internet platforms. This may explain the relatively large proportion of patients aged under 40 years and patients with MS as a primary etiology for their spasticity, as these patients have been shown to be well-informed and engaged in their condition management, and thus are very active on these platforms [46]. Patients with stroke are typically older (aged >65 years) and may be less likely to engage with online surveys. In addition, it should be considered that caregiver burden may vary with etiology. As discussed, MS was the most common cause of spasticity in this study; however, strokes typically affect older populations and, consequently, patients may have older caregivers, which could potentially cause a higher degree of burden. In this study, there was an age range of 4.1 years between the caregivers of patients with stroke and MS, which does not suggest a greater degree of burden on caregivers of patients with stroke due to older age. However, this small age range may be reflective of the social media aspect of this system, as mentioned previously, rather than the real-world situation. Other limitations were the lack of severity assessment of the patients' spasticity, and that data reflect patients' and caregivers' perceptions of treatment effectiveness rather than accepted clinical endpoints. Additionally, a large proportion of patients received concomitant physiotherapy and oral medications during treatment with BoNT-A; as a result of the nature of this patient survey, it is not possible to deduce how these confounding factors affected patients' and caregivers' perceptions of the efficacy of BoNT-A treatment.

### Conclusions

From the patient and caregiver's perspectives elucidated in this study, spasticity represents a great burden on many aspects of their lives, including the ability to work, QoL, and difficulties with daily living. Several challenges were identified with receiving BoNT-A treatment for spasticity, predominantly around scheduling and the associated costs of injections. Despite these challenges, it was established that patients and caregivers perceive that BoNT‑A improves patients' lives, with high levels of overall satisfaction reported. However, patients and caregivers reported that providing efficacy was maintained, BoNT-A injections of reduced frequency would alleviate practical issues associated with treatment and, more importantly, provide prolonged symptom relief. Using an online community enabled more rapid recruitment to the study and data collection regarding patient and caregiver perspectives than would have been possible in a clinical trial or a validation study of patient-reported outcomes.

## Appendix

Multimedia Appendix 1 Questionnaire.

Multimedia Appendix 2 Participant characteristics in the European Union and the United States.

## Abbreviations

BoNT-A: botulinum neurotoxin type A
CI: confidence interval
CNS: central nervous system
FDA: US Food and Drug Administration
MS: multiple sclerosis
QoL: quality of life
