# Supplementary material for: Perspective of an International Online Patient and Caregiver Community on the Burden of Spasticity and Impact of Botulinum Neurotoxin Therapy: Survey Study
Source: JMIR Public Health Surveill. 2020 Dec 7;6(4):e17928. doi: 10.2196/17928 (PMC7752537; doi:10.2196/17928)
Supplement: Multimedia Appendix 1 [file publichealth_v6i4e17928_app1.docx]

**Description message:**

Hello,

We invite you to participate in an international survey designed **to better understand patients’ and caregivers’ experience with Botulinum Toxin A injections**. These injections are given to deal with symptoms related with spasticity which could include muscle spasms, rigidity and pain, unwanted movement of the stiff limb, difficulty using the arms or/and the legs…

As a patient or as a caregiver, your feedback about symptoms and treatments will be useful to better understand your needs and how we can improve treatments and solutions.

Your answers will remain strictly **anonymous**, and will only be reported in **aggregate** (no individual answers will be shared). They will be confidential. You may withdraw at any time. We will share a summary of the results with all participants.

This international study is conducted in partnership with an international team of 4 medical experts and a pharmaceutical company that manufactures a product approved for the treatment of spasticity.

Thank you in advance for your time and much appreciated contribution!

The Carenity Team

## **Black: to all respondents**

**Blue: to patients only**
**Green: to caregivers only**

## **A. Respondent’s profile**

1. **You are a:**

*(Single answer)*

- Man
- Woman
- Transgender

1. **Your date of birth:**

(mm/yyyy) [SCREEN OUT if age<18 y/o]

1. **Your country of residence:**

*(Single answer)*

- France (overseas territories included)
- Germany
- Italy
- Spain
- UK (England, Wales, Scotland, Northern Ireland)
- USA
- Other*[Specify]* [SCREEN OUT]

1. **Are you affected / were you affected by one of the following conditions?**

*(Single answer)*

- Multiple sclerosis
- Stroke
- Traumatic brain injury
- Spinal cord injury
- Cerebral palsy
- Brain tumour
- Spastic paraplegia
- No, but I am the caregiver of a patient affected by one of the previous conditions >Q4bis
- No, I am not affected by any of these conditions [SCREEN OUT]

**4bis. Please tell us which condition the patient you take care of is affected by / was affected by.**

*(Single answer)*

- Multiple sclerosis
- Stroke
- Traumatic brain injury
- Spinal cord injury
- Cerebral palsy
- Brain tumour
- Spastic paraplegia
- None of the above conditions [SCREEN OUT]

4ter. On average, how often do you take care of the patient?

*(Single answer)*

- Less than once a week
- At least 1 day a week
- At least 2 days a week
- At least 4 days a week
- Everyday

**4qater. When did you start taking care of the patient?**

*(If you do not remember the month,, please give us your best estimate.)*

(mm/yyyy)

1. **What is your relationship with the patient?**

*(Single answer)*

- The patient is my partner
- The patient is my child
- The patient is my mother/my father
- The patient is my brother/sister
- The patient is another member of my family
- The patient is my friend
- The patient is my neighbour
- Other *[Specify]*

1. **The patient you care for is a:**

*(Single answer)*

- Man
- Woman

1. **What is the date of birth of the patient you care for?**

(mm/yyyy) [SCREEN OUT if age<18 y/o]

1. **When were you diagnosed with [ANSWER_Q4]?**

**8bis. When was the patient diagnosed with [ANSWER_Q4bis]?**

*(If you do not remember the month, please give us your best estimate.)*

(mm/yyyy)

1. **Which of the following symptoms have you experienced/are you experiencing as a consequence of [ANSWER_Q4]?**

**9bis. Which of the following symptoms has the patient experienced/is experiencing as a consequence of his/her [ANSWER_Q4bis]?**

*(Multiple answers)*

- Muscle spasms
- Muscle stiffness/rigidity (including painful contractures)
- Muscle pain
- Unwanted movement of the stiff limb
- Difficulties using my legs(i.e. falling, tripping, loss of balance)
- Difficulties using my arm(s) (extending my arms, opening my hands…)
- None of the above [SCREEN OUT]

**INSERT:**

In the following questions, we will use the word **“spasticity”** to refer to the symptoms you selected in the previous question among **muscle spasms, muscle stiffness/rigidity, muscle pain, unwanted movement of the stiff limb, difficulties using the legs** (i.e. falling, tripping, loss of balance), **or difficulties using the arms** *(extending the arms, opening the arms…)***.**

1. **In which limbs do you experience spasticity symptoms?**

**10bis. In which limbs does the patient experience spasticity symptoms?**

*Spasticity refers to one or several of the following symptoms: muscle spasms, muscle stiffness/rigidity, muscle pain, unwanted movement of the stiff limb, difficulties using the legs (i.e. falling, tripping, loss of balance) or difficulties using the arms (extending the arms, opening the arms…).*

*(Multiple answers)*

- Right arm
- Left arm
- Right leg
- Left leg

1. **Which treatments are you currently receiving for your spasticity?**

**11bis. Which treatments is the patient currently receiving for his/her spasticity?**

*Spasticity refers to one or several of the following symptoms: muscle spasms, muscle stiffness/rigidity, muscle pain, unwanted movement of the stiff limb, difficulties using the legs (i.e. falling, tripping, loss of balance) or difficulties using the arms (extending the arms, opening the arms…).*

*(Multiple answers)*

- Oral medication (muscle relaxant, oral baclofen…)
- Botulinum toxin A injections (injections into the muscle: Botox, Dysport, Xeomin)
- Botulinum toxin B injections (injections into the muscle: MyoBloc, NeuroBloc)
- Phenol injections
- Alcohol injections
- Intrathecal baclofen therapy
- Physiotherapy at home
- Physiotherapy at hospital/clinic
- Home based self-rehabilitation
- Other *[Specify]*
- I do not take any specific treatment for my spasticity

**[Respondents who did not select one of the Botulinum Toxin A treatments will be screened out.]**

1. **What is the name of the Botulinum Toxin A injections you are receiving for your spasticity?**

**12bis. What is the name of the Botulinum Toxin A injections the patient is receiving for his/her spasticity?**

*Spasticity refers to one or several of the following symptoms: muscle spasms, muscle stiffness/rigidity, muscle pain, unwanted movement of the stiff limb, difficulties using the legs (i.e. falling, tripping, loss of balance) or difficulties using the arms (extending the arms, opening the arms…).*

- Botox
- Dysport
- Xeomin
- I do not know

1. **When did you start receiving Botulinum Toxin A injections?**

**13bis. When did the patient start receiving Botulinum Toxin A injections?**

*(If you do not remember the month, please give us your best estimate.)*

(mm/yyyy) [SCREEN OUT if <1 year]

## **B. Impact of spasticity functioning, on quality of life and work productivity**

1. **Please tell us about your employment status.**

**Full time: more than 30 hours per week*

**Part-time: less than 30 hours per week*

*(Single answer)*

- I work full time*
- I work part-time* (less than 30 hours per week) because of my condition
- I work part-time* but it is not due to my condition
- I do not work because of my condition
- I do not work but it is not due to my condition (retired, …)
- I am a full time student
- Other *[Specify]*

1. **Please tell us about your employment status as a caregiver.**

**Full time: more than 30 hours per week*

**Part-time: less than 30 hours per week*

*(Single answer)*

- I work full time
- I work part-time because I need time to take care of the patient
- I work part-time but it is not due to the patient’s condition
- I do not work because I need time to take care of the patient
- I do not work but it is not due to the patient’s condition (retired, housewife…)
- I am a full time student
- Other *[Specify]*

1. **Has your condition affected someone else’s professional life?**

*(Single answer)*

- Yes, someone had to change his/her professional life to take care of me
- Yes, someone had to stop working to take care of me
- No

1. **For each of the following items, please assess the level of difficulty you experience due to your spasticity.**

**17bis. For each of the following items, please assess the level of difficulty the patient you take care of experiences due to his/her spasticity.**

*Spasticity refers to one or several of the following symptoms: muscle spasms, muscle stiffness/rigidity, muscle pain, unwanted movement of the stiff limb, difficulties using the legs(i.e. falling, tripping, loss of balance) or difficulties using the arms (extending the arms, opening the arms…).*

*(1 answer per line, 0=no disability, 10=severe disability)*

|  | **0**  **No difficulty** | **1** | **2** | **3** | **4** | **5** | **6** | **7** | **8** | **9** | **10**  **Great Difficulty** |
| --- | --- | --- | --- | --- | --- | --- | --- | --- | --- | --- | --- |
| *Difficulty to perform daily tasks (e.g. prepare meals, groom yourself, dress…)* | o | o | o | o | o | o | o | o | o | o | o |
| *Difficulty to use a computer* | o | o | o | o | o | o | o | o | o | o | o |
| *Difficulty to write* | o | o | o | o | o | o | o | o | o | o | o |
| *Difficulty to walk* | o | o | o | o | o | o | o | o | o | o | o |
| *Diificulty to drive* | o | o | o | o | o | o | o | o | o | o | o |
| *Difficulty to carry something* | o | o | o | o | o | o | o | o | o | o | o |
| *Difficulty to catch something* | o | o | o | o | o | o | o | o | o | o | o |

1. **Please assess to what extent spasticity affects your life.**

*Spasticity refers to one or several of the following symptoms: muscle spasms, muscle stiffness/rigidity, muscle pain, unwanted movement of the stiff limb, difficulties using the legs(i.e. falling, tripping, loss of balance) or difficulties using the arms (extending the arms, opening the arms…).*

*(1 answer per line, 0=no impact, 10=a great impact)*

|  | **0**  **No impact** | **1** | **2** | **3** | **4** | **5** | **6** | **7** | **8** | **9** | **10**  **Greatly impacted** |
| --- | --- | --- | --- | --- | --- | --- | --- | --- | --- | --- | --- |
| *Relationship with family and friends* | o | o | o | o | o | o | o | o | o | o | o |
| *Leisure* | o | o | o | o | o | o | o | o | o | o | o |
| *Depression and mood alterations* | o | o | o | o | o | o | o | o | o | o | o |
| *Self-esteem* | o | o | o | o | o | o | o | o | o | o | o |
| *Lack of sleep/Fatigue* | o | o | o | o | o | o | o | o | o | o | o |
| *Professional life* | o | o | o | o | o | o | o | o | o | o | o |
| *Sexual life* | o | o | o | o | o | o | o | o | o | o | o |
| *Impact on overall quality of life* | o | o | o | o | o | o | o | o | o | o | o |

**18bis. Please assess to what extent spasticity affects the patient’s life.**

*Spasticity refers to one or several of the following symptoms: muscle spasms, muscle stiffness/rigidity, muscle pain, unwanted movement of the stiff limb, difficulties using the legs(i.e. falling, tripping, loss of balance) or difficulties using the arms (extending the arms, opening the arms…).*

*(1 answer per line, 0=no impact, 10=a great impact)*

|  | **0**  **No impact** | **1** | **2** | **3** | **4** | **5** | **6** | **7** | **8** | **9** | **10**  **Greatly impacted** |
| --- | --- | --- | --- | --- | --- | --- | --- | --- | --- | --- | --- |
| *Relationship with family and friends* | o | o | o | o | o | o | o | o | o | o | o |
| *Leisure* | o | o | o | o | o | o | o | o | o | o | o |
| *Depression and mood alterations* | o | o | o | o | o | o | o | o | o | o | o |
| *Self-esteem* | o | o | o | o | o | o | o | o | o | o | o |
| *Lack of sleep/Fatigue* | o | o | o | o | o | o | o | o | o | o | o |
| *Professional life* | o | o | o | o | o | o | o | o | o | o | o |
| *Sexual life* | o | o | o | o | o | o | o | o | o | o | o |
| *Impact on overall quality of life* | o | o | o | o | o | o | o | o | o | o | o |

## **C. Impact of injections on quality of life**

1. **Have you discussed the goals of Botulinum Toxin A treatment with your doctor?**

*(Single answer)*

- Yes
- No

**19bis. Were the goals of Botulinum Toxin A treatment discussed with you and/or the patient you take care of?**

*(Single answer)*

- Yes
- No
- I do not know

1. **Do you know what the time interval between each Botulinum Toxin A treatment is for the patient you take care of?**

*(Single answer)*

- Yes
- No >Q21bis

**20bis. On average, how many Botulinum Toxin A treatments do you receive per year?**

**20ter. On average, how many Botulinum Toxin A treatments does the patient receive per year?**

*(Minimum=1, maximum=12)*

Numeric

1. **Do you plan the next treatment date immediately after you get your injections of Botulinum Toxin A with your doctor ?**

*Please select the answer which best describes your situation.*

*(Single answer)*

- Yes, the next appointment is planned immediately as the interval between two injections is always the same
- Yes, the next appointment is planned immediately but has sometimes to be arranged for an earlier date depending on my spasticity symptoms
- Yes, the next appointment is planned immediately, but I would like it to be arranged earlier depending on my spasticity symptoms, but that is not possible (due to insurance…)
- No, the interval between injections is not regular and the appointment with the doctor is arranged when needed
- Other *[Specify]*

**21bis. Is the next treatment date planned immediately after the patient gets his/her injections of Botulinum Toxin A with his/her doctor?**

*Please select the answer which best describes your situation.*

*(Single answer)*

- Yes, the next appointment is planned immediately as the interval between two injections is always the same
- Yes, the next appointment is planned immediately but has sometimes to be arranged earlier depending on his/her spasticity symptoms
- Yes, the next appointment is planned immediately, but he/she would like it to be arranged for an earlier date depending on his/her spasticity symptoms, but that is not possible (due to insurance…)
- No, the interval between injections is not regular and the appointment with the doctor is arranged when needed
- Other *[Specify]*
- I do not know

1. **Please assess to what extent your current Botulinum Toxin A injections improve your life.**

**22bis. Please assess to what extent the patient’s current Botulinum Toxin A injections improve his/her life.**

*(1 answer per line, 0 = no improvement, 10 = a great improvement)*

|  | **0**  **No improvement** | **1** | **2** | **3** | **4** | **5** | **6** | **7** | **8** | **9** | **10**  **Greatly improved** |
| --- | --- | --- | --- | --- | --- | --- | --- | --- | --- | --- | --- |
| *Muscle stiffness/muscle spasm* | o | o | o | o | o | o | o | o | o | o | o |
| *Pain in arms and/or legs* | o | o | o | o | o | o | o | o | o | o | o |
| *Ability to perform daily tasks (e.g. prepare meals, groom yourself, dress…)* | o | o | o | o | o | o | o | o | o | o | o |
| *Ability to walk* | o | o | o | o | o | o | o | o | o | o | o |
| *Transfers (moving around, short trips)* | o | o | o | o | o | o | o | o | o | o | o |
| *Lack of sleep/Fatigue* | o | o | o | o | o | o | o | o | o | o | o |
| *Self-confidence* | o | o | o | o | o | o | o | o | o | o | o |
| *Leisure* | o | o | o | o | o | o | o | o | o | o | o |
| *Relationship with family and friends* | o | o | o | o | o | o | o | o | o | o | o |
| *Ability/desire to socialize (approach others)* | o | o | o | o | o | o | o | o | o | o | o |
| *Willingness to perform activities* | o | o | o | o | o | o | o | o | o | o | o |
| *Depression and mood alterations* | o | o | o | o | o | o | o | o | o | o | o |
| *Professional life* | o | o | o | o | o | o | o | o | o | o | o |
| *Sexual life* | o | o | o | o | o | o | o | o | o | o | o |
| *Anxiety about the future* | o | o | o | o | o | o | o | o | o | o | o |
| *Overall satisfaction* | o | o | o | o | o | o | o | o | o | o | o |

1. **What are your main issues or concerns about Botulinum Toxin A injections?**

Please share what is on your mind.

Detail your answer as much as possible.

*Text*

1. **Please assess the burden of receiving Botulinum Toxin A injections:**

(1 answer per li*ne,* 0 = it is not a problem at all, 10 = it is very problematic)

|  | **0**  **No problem** | **1** | **2** | **3** | **4** | **5** | **6** | **7** | **8** | **9** | **10**  **Very problematic** |
| --- | --- | --- | --- | --- | --- | --- | --- | --- | --- | --- | --- |
| *Fear of injections or needle* | o | o | o | o | o | o | o | o | o | o | o |
| *Pain during/after injections* | o | o | o | o | o | o | o | o | o | o | o |
| *Frequency of injections* | o | o | o | o | o | o | o | o | o | o | o |
| *Availability of timely appointments/someone to perform the injections* | o | o | o | o | o | o | o | o | o | o | o |
| *Logistics (travel to hospital, time spent on injections…)* | o | o | o | o | o | o | o | o | o | o | o |
| *Cost of getting injections* | o | o | o | o | o | o | o | o | o | o | o |

**[Q25 is only for respondents who work part-time or full time]**

1. **Do you have to take time off work (e.g. full day, half day, several hours) to get your Botulinum Toxin A injections?**

**25bis. Do you have to take time off work (e.g. full day, half day, several hours) to go with the patient to get his/her Botulinum Toxin A injections?**

*Please select the answer which best describes your situation.*

*(Single answer)*

- No, never **>Q27**
- Yes, sometimes
- Yes, often
- Yes, always

1. **On average, how many days off per year do you have to take to get your Botulinum Toxin A injections?**

**26bis. On average, how many days off per year do you have to take to go with the patient to get his/her Botulinum Toxin A injections?**

*(Minimum=0)*

Numeric field

1. **When you get your Botulinum Toxin A injections, which of the following costs do you have to pay?**

**27bis. When the patient gets his/her Botulinum Toxin A injections, which of the following costs do you have to pay as a caregiver?**

*(Multiple answers)*

- Transportation costs
- Parking costs
- Out-of-pocket expenses related to office visits/consultations
- Out-of-pocket expenses related to treatment (Botulinum Toxin A injections)
- Reduced salary due to missing work hours
- Other *[Specify]*
- None **Q>29**

1. **Please indicate what you estimate is the total financial cost each time you get your injections, in [currency].**

**28bis. Please indicate what you estimate is your total financial cost each time the patient gets his/her injections, in [currency].**

*(Minimum=0)*

Numeric field

## **D. Impact of longer time to retreatment**

**INSERT:**

In the following questions, we assume that the effect of Botulinum Toxin A injections is lasting longer while the onset of action does not vary.

1. **Assuming the effect of Botulinum Toxin A injections could last longer, would you see any benefits of having less frequent injections?**

*(Single answer)*

- Yes, many
- Yes, some
- Yes, a few
- No **>Q31**

1. **Assuming the effect of Botulinum Toxin A injections could last longer, what would less frequent Botulinum Toxin A injections mean to you?**

How would that impact your life? Your state of mind? etc.

Please detail your answer as much as possible.

*Text*

1. **Assuming the effect of Botulinum Toxin A injections could last longer and you had less frequent injections, what would be the 3 most important benefits for you?**

*(Up to 3 answers, randomised items)*

- Longer periods not worrying about spasticity symptoms
- Reliving my fear for injections less frequently
- Less impact on work activities (days off…)
- Less logistics burden (organisation, transport, time spent…)
- More quality time with family and friends
- Less dependence on others/caregivers
- More self-confidence
- More self-esteem
- Longer periods with improved mobility
- Other *[Specify]*
- I would not experience any benefits

**31bis. Assuming the effect of Botulinum Toxin A injections could last longer and the patient had less frequent injections, what would be the 3 most important benefits for you?**

*(Up to 3 answers, randomised items)*

- Longer periods not worrying about spasticity symptoms
- Less impact on work activities (days off…)
- Less logistics burden (organisation, transport, time spent…)
- More quality time with family and friends
- Other *[Specify]*
- I would not experience any benefits

1. **Assuming the effect of Botulinum Toxin A injections could last longer, with how many injections per year would you feel the benefit of less frequent injections?**

*For example, if you currently receive 4 injections per year and you think you would need 3 injections per year to feel the benefit of less frequent injections, you will write 3 below.*

*(Minimum=0, maximum=12)*

Numeric

1. **Assuming the effect of Botulinum Toxin A injections could last longer and you had less frequent injections, please assess how it would improve the burden of Botulinum Toxin A injections.***For example, you would receive injections for your spasticity 3 times a year instead of 4.*

*(1 answer per line, 0=no improvement, 10=it would improve greatly)*

|  | **0**  **No improvement** | **1** | **2** | **3** | **4** | **5** | **6** | **7** | **8** | **9** | **10**  **Greatly improved** |
| --- | --- | --- | --- | --- | --- | --- | --- | --- | --- | --- | --- |
| *Fear of injections or needle* | o | o | o | o | o | o | o | o | o | o | o |
| *Pain during/after injections* | o | o | o | o | o | o | o | o | o | o | o |
| *Availability of timely appointments/someone to perform the injections* | o | o | o | o | o | o | o | o | o | o | o |
| *Logistics (travel to hospital, time spent on injections…)* | o | o | o | o | o | o | o | o | o | o | o |
| *Cost of getting injections* | o | o | o | o | o | o | o | o | o | o | o |
| *General impact on quality of life* | o | o | o | o | o | o | o | o | o | o | o |

**33bis. Assuming the effect of Botulinum Toxin A injections could last longer and the patient had less frequent injections~~,~~ please assess how it would improve the burden of Botulinum Toxin A injections for you.**

*For example, the patient would receive injections for his/her spasticity 3 times a year instead of 4.*

*(1 answer per line, 0=no improvement, 10=it would improve greatly)*

|  | **0**  **No improvement** | **1** | **2** | **3** | **4** | **5** | **6** | **7** | **8** | **9** | **10**  **Greatly improved** |
| --- | --- | --- | --- | --- | --- | --- | --- | --- | --- | --- | --- |
| *Availability of timely appointments/someone to perform the injections* | o | o | o | o | o | o | o | o | o | o | o |
| *Logistics (travel to hospital, time spent on injections…)* | o | o | o | o | o | o | o | o | o | o | o |
| *Cost of getting injections* | o | o | o | o | o | o | o | o | o | o | o |
| *General impact on quality of life* | o | o | o | o | o | o | o | o | o | o | o |

1. **Assuming the effect of Botulinum Toxin A injections could last longer and you had less frequent injections, how would you feel about a longer period of time between each medical visit?** *(Single answer)*

- I would not mind having less frequent medical visits
- I would not feel comfortable with less frequent medical visits
- I would arrange appointments even if injections were not needed to see my doctor more frequently
- Other *[Specify]*
- Less frequent injections would not reduce medical visits frequency

We thank you for taking the time to participate in this survey on spasticity.

We will share a synthesis of the survey results with all the participants.

You can talk about your experience with other Carenity members **in this discussion**!

Your opinion matters!

See you soon,

The Carenity Team
