# Supplementary material for: Perspective of an International Online Patient and Caregiver Community on the Burden of Spasticity and Impact of Botulinum Neurotoxin Therapy: Survey Study
Source: JMIR Public Health Surveill. 2020 Dec 7;6(4):e17928. doi: 10.2196/17928 (PMC7752537; doi:10.2196/17928)
Supplement: Multimedia Appendix 2 [file publichealth_v6i4e17928_app2.docx]

**Participant characteristics in the European Union and the United States.**

|  | **Europe**  **(n=315)** | **USA**  **(n=300)** | **Total population**  **(n=615)** |
| --- | --- | --- | --- |
| **Cohort, n (%)** | 315 (51) | 300 (49) | 615 (100) |
| Patient | 249 (79) | 178 (59) | 427 (69) |
| Caregiver/caregiver’s patient | 66 (21) | 122 (41) | 188 (31) |
| **Age of patients, n (%)** | n=249 | n=178 | n=427 |
| 18-30 years | 38 (15) | 34 (19) | 72 (17) |
| 31-40 years | 52 (21) | 75 (42) | 127 (30) |
| 41-50 years | 86 (35) | 48 (27) | 134 (31) |
| ≥51 years | 73 (29) | 21 (11) | 94 (22) |
| **Age of caregivers, n (%)** | n=66 | n=122 | n=188 |
| 18-30 years | 22 (33) | 27 (22) | 49 (26) |
| 31-40 years | 18 (27) | 45 (37) | 63 (34) |
| 41-50 years | 15 (23) | 30 (25) | 45 (24) |
| ≥51 years | 11 (17) | 20 (16) | 31 (16) |
| **Age of caregivers’ patients, n (%)** | n=66 | n=122 | n=188 |
| 18-30 years | 9 (14) | 15 (12) | 24 (13) |
| 31-40 years | 4 (6) | 11 (9) | 15 (8) |
| 41-50 years | 12 (18) | 13 (11) | 25 13) |
| ≥51 years | 41 (62) | 83 (68) | 124 (66) |
| **Sex of patients, n (%)** | n=249 | n=178 | n=427 |
| Male | 134 (54) | 82 (46) | 216 (51) |
| Female | 113 (45) | 93 (52) | 206 (48) |
| Transgender | 2 (1) | 3 (2) | 5 (1) |
| **Sex of caregiver, n (%)** | n=66 | n=122 | n=188 |
| Male | 32 (48) | 52 (43) | 84 (45) |
| Female | 34 (52) | 70 (57) | 104 (55) |
| Transgender | 0 (0) | 0 (0) | 0 (0) |
| **Sex of caregivers’ patients, n (%)** | n=66 | n=122 | n=188 |
| Male | 33 (50) | 51 (42) | 84 (45) |
| Female | 33 (50) | 71 (58) | 104 (55) |
| Transgender | 0 (0) | 0 (0) | 0 (0) |
| **Relationship of patient to caregiver, n (%)** | n=66 | n=122 | n=188 |
| Parent | 24 (36) | 52 (43) | 76 (40) |
| Another family member | 22 (33) | 29 (24) | 51 (27) |
| Friend | 4 (6) | 13 (11) | 17 (9) |
| Partner | 5 (8) | 9 (7) | 14 (7) |
| Child | 4 (6) | 7 (6) | 11 (6) |
| Sibling | 4 (6) | 7 (6) | 11 (6) |
| Neighbor | 2 (3) | 4 (3) | 6 (3) |
| Other | 1 (2) | 1 (1) | 2 (1) |
| **Frequency of caregiving, n (%)** | n=66 | n=122 | n=188 |
| ≥1 day a week | 8 (12) | 6 (5) | 14 (7) |
| ≥2 days a week | 14 (21) | 13 (11) | 27 (14) |
| ≥4 days a week | 17 (26) | 45 (37) | 62 (33) |
| Daily | 27 (41) | 58 (48) | 85 (45) |
| **Duration of caregiving, n (%)** | n=66 | n=122 | n=188 |
| <1 year | 3 (5) | 8 (7) | 11 (6) |
| 1-3 years | 22 (33) | 42 (34) | 64 (34) |
| 3-5 years | 12 (18) | 34 (28) | 46 (24) |
| 5-10 years | 21 (32) | 24 (20) | 45 (24) |
| >10 years | 8 (12) | 14 (11) | 22 (12) |
| **Cause of spasticity (patients/caregivers), n (%)** | n=249/66 | n=178/122 | n=615 |
| Brain tumor | 8 (3)/1 (2) | 5 (3)/4 (3) | 18 (3) |
| Cerebral palsy | 15 (6)/9 (14) | 16 (9)/10 (8) | 50 (8) |
| Multiple sclerosis | 134 (54)/20 (30) | 65 (37)/37 (30) | 256 (42) |
| Spastic paraplegia | 10 (4)/8 (12) | 31 (17)/12 (10) | 61 (10) |
| Spinal cord injury | 16 (6)/6 (9) | 24 (13)/14 (11) | 60 (10) |
| Stroke | 46 (18)/17 (26) | 23 (13)/36 (30) | 122 (20) |
| Traumatic brain injury | 20 (8)/5 (8) | 14 (8)/9 (7) | 48 (8) |
| **Time since diagnosis (patients/caregivers), n (%)** | n=249/66 | n=178/122 | n=427/188 |
| <3 years | 64 (25)/14 (21) | 76 (43)/30 (25) | 140 (33)/44 (23) |
| 3-5 years | 35 (14)/12 (18) | 26 (15)/30 (25) | 61 (14)/42 (22) |
| 5-10 years | 49 (20)/23 (35) | 30 (17)/34 (28) | 79 (19)/57 (30) |
| 10-15 years | 40 (16)/3 (5) | 14 (8)/7 (6) | 54 (13)/10 (5) |
| >15 years | 52 (21)/13 (20) | 22 (12)/17 (14) | 74 (17)/30 (16) |
| Not specified | 9 (4)/1 (2) | 10 (6)/4 (3) | 19 (4)/5 (3) |
| **Limbs affected (patients/caregivers), n (%)** | n=249/66 | n=178/122 | n=427/188 |
| Left arm | 137 (55)/42 (64) | 100 (56)/58 (48) | 237 (56)/100 (53) |
| Left leg | 126 (51)/36 (55) | 93 (52)/61 (50) | 219 (51)/97 (52) |
| Right arm | 131 (53)/34 (52) | 106 (60)/75 (61) | 237 (56)/109 (58) |
| Right leg | 149 (60)/40 (61) | 117 (66)/75 (61) | 266 (62)/115 (61) |
| Lower limbs only | 52 (21)/9 (14) | 31 (17)/19 (16) | 83 (19)/28 (15) |
| Upper limbs only | 56 (22)/11 (17) | 34 (19)/24 (20) | 90 (21)/35 (19) |
| Right arm and leg | 35 (14)/10 (15) | 27 (15)/26 (21) | 62 (15)/36 (19) |
| Left arm and leg | 28 (11)/12 (18) | 12 (7)/16 (13) | 40 (9)/28 (15) |
| Diagonal only | 13 (5)/1 (2) | 14 (8)/4 (3) | 27 (6)/5 (3) |
| >2 limbs | 65 (26)/23 (35) | 60 (34)/33 (27) | 125 (29)/56 (30) |
| **Symptoms experienced (patients/caregivers), n (%)** | n=249/66 | n=178/122 | n=427/188 |
| Difficulties using arm/s | 101 (41)/38 (58) |  |  |
| Difficulties using legs | 164 (66)/45 (68) | 93 (52)/70 (57) | 194 (45)/108 (57) |
| Muscle pain | 163 (65)/49 (74) | 122 (69)/88 (72) | 286 (67)/133 (71) |
| Muscle spasms | 171 (69)/41 (62) | 132 (74)/80 (66) | 295 (69)/129 (69) |
| Muscle stiffness/rigidity | 166 (67)/51 (77) | 137 (77)/84 (69) | 308 (72)/125 (66) |
| Unwanted movement of the stiff limb | 97 (39)/26 (39) | 129 (72)/85 (70) | 295 (69)/136 (72) |
| **Total number of symptoms experienced  (patients/caregivers), n (%)** | n=249/66 | n=178/122 | n=427/188 |
| 1 | 19 (8)/2 (3) | 12 (7)/7 (6) | 31 (7)/9 (5) |
| 2 | 47 (19)/10 (15) | 24 (13)/17 (14) | 71 (17)/27 (14) |
| 3 | 71 (29)/16 (24) | 29 (16)/34 (28) | 100 (23)/50 (27) |
| 4 | 55 (22)/22 (33) | 51 (29)/31 (25) | 106 (25)/53 (28) |
| ≥5 | 57 (23)/16 (24) | 62 (35)/33 (27) | 119 (28)/49 (26) |
| **BoNT-A treatment received^a^ (patients/caregivers), n (%)** | n=249/66 | n=178/122 | n=427/188 |
| AbobotulinumtoxinA | 41 (16)/10 (15) | 34 (19)/24 (20) | 75 (18)/34 (18) |
| IncobotulinumtoxinA | 24 (10)/7 (11) | 24 (13)/12 (10) | 48 (11)/19 (10) |
| OnabotulinumtoxinA | 128 (51)/24 (36) | 109 (61)/77 (63) | 237 (56)/101 (54) |
| Other^b^ | 3 (1)/0 (0) | 0 (0)/0 (0) | 3 (1)/0 (0) |
| Do not know | 53 (21)/25 (38) | 11 (6)/9 (7) | 64 (15)/34 (18) |
| **Time since treatment initiation (patients/caregivers), n (%)** | n=249/66 | n=178/122 | n= 427/188 |
| <2 years | 107 (43)/19 (29) | 96 (54)/49 (40) | 203 (48)/68 (36) |
| 2-5 years | 68 (27)/24 (36) | 50 (28)/44 (36) | 118 (28)/68 (36) |
| 5-10 years | 40 (16)/16 (24) | 21 (12)/24 (20) | 61 (14)/40 (21) |
| 10-15 years | 22 (9)/6 (9) | 7 (4)/2 (2) | 29 (7)/8 (4) |
| >15 years | 12 (5)/1 (2) | 4 (2)/3 (2) | 16 (4)/4 (2) |
| Mean time since treatment initiation, years (95% CI) | 4.0 (3.4;4.7)/4.0 (3.1;4.9) | 2.6 (2.1;3.2)/3.2 (2.5;4.0) | 3.5 (3.0;3.9)/3.5 (2.9;4.1) |
| **Concomitant therapy (patients/caregivers), n (%)** | n=249/66 | n=178/122 | n=427/188 |
| Injections |  |  |  |
| Alcohol | 16 (6)/7 (11) | 13 (7)/1 (1) | 29 (7)/8 (4) |
| Botulinum B | 24 (10)/8 (12) | 16 (9)/16 (13) | 40 (9)/24 (13) |
| Intrathecal baclofen | 24 (10)/14 (21) | 13 (7)/3 (2) | 37 (9)/17 (9) |
| Phenol | 27 (11)/8 (12) | 18 (10)/5 (4) | 45 (11)/13 (7) |
| Oral medications^c^ | 150 (60)/37 (56) | 106 (60)/72 (59) | 256 (60)/109 (58) |
| Physiotherapy at home | 93 (37)/33 (50) | 58 (33)/45 (37) | 151 (35)/78 (41) |
| Physiotherapy at hospital/clinic | 84 (34)/28 (42) | 62 (35)/28 (23) | 146 (34)/56 (30) |
| Self-rehabilitation (home-based) | 43 (17)/23 (35) | 36 (20)/42 (34) | 79 (19)/65 (35) |
| Other | 2 (1)/0 (0) | 0 (0)/0 (0) | 2 (0)/0 (0) |

^a^Self-reported.

^b^For respondents from Spain only, brand names given were Bocouture, Lantox, Azzalure.

^c^Eg, muscle relaxants, baclofen.

BoNT-A = botulinum neurotoxin type A.
